# Physicochemical Characterization, In Vitro Anti-Aging Enzyme Modulation, and Dermocosmetic Application of *Prunus spinosa* L. Kernel Oil

**DOI:** 10.3390/molecules31040632

**Published:** 2026-02-12

**Authors:** Asmaa Berkati, Nadir Ben Hamiche, Louiza Himed, Lynda Guerboub, Salah Merniz, Maria D’Elia, Luca Rastrelli

**Affiliations:** 1 Université de Bejaia, Faculté des Sciences de la Nature Et de la Vie, Laboratoire de Biomathématiques, Biochimie,Biophysique Et Scientométrie (L3BS), Biophysique Et Scientométrie (L3BS) , Bejaia 06000, Algerianadir.benhamiche@univ-bejaia.dz (N.B.H.);; 2 Laboratory of Biotechnology and Food Quality (BIOQUAL), Institute of Nutrition, Food and Agri-Food Technologies (INATAA), University of Constantine 1, Constantine 25000, Algeria; 3 Institute of Industrial Hygiene and Safety, University Batna 2, Batna 05000, Algeria; 4 Department of Pharmacy, University of Salerno, Via Giovanni Paolo II, 132, 84084 Salerno, Italy; 5 National Biodiversity Future Center (NBFC), 90133 Palermo, Italy; 6 Dipartimento di Scienze della Terra e del Mare, University of Palermo, 90123 Palermo, Italy

**Keywords:** *Prunus spinosa* L., kernel oil, dermocosmetic applications, anti-aging enzymes, matrix metalloproteinase-9, collagenase, UV-protective properties

## Abstract

*Prunus spinosa* L. kernel oil (PSKO) is an underexplored natural oil rich in unsaturated fatty acids and minor bioactive constituents with potential dermocosmetic relevance. In this study, PSKO was comprehensively characterized in terms of physicochemical quality parameters, fatty acid composition, phenolic and carotenoid content, and antioxidant capacity. Gas chromatography–mass spectrometry (GC–MS) analysis revealed oleic acid (65.9%) and linoleic acid (18.8%) as the predominant fatty acids. The biological potential of PSKO was further investigated through in vitro anti-aging enzyme assays, showing a concentration-dependent inhibition of key extracellular matrix-degrading enzymes, including matrix metalloproteinase-9 (MMP-9), collagenase, and elastase, thus indicating a multi-target modulation of enzymatic pathways involved in skin aging. Based on its chemical composition and bioactivity profile, PSKO was incorporated into a moisturizing cream formulation. The PSKO-enriched cream exhibited improved physicochemical stability, enhanced occlusive properties, and higher sensory acceptability compared with the control formulation. In addition, UV–visible spectrophotometric analysis demonstrated that both PSKO and the PSKO-enriched cream displayed measurable absorption in the ultraviolet B (UVB) and ultraviolet A (UVA) regions, together with good photostability, supporting a complementary in vitro UV-protective role. Overall, these findings highlight PSKO as a sustainable and multifunctional bioactive ingredient derived from an underutilized agri-food by-product, with promising potential for innovative dermocosmetic applications.

## 1. Introduction

The rapid growth of the food industry and the intensification of food processing technologies have resulted in a substantial increase in by-product generation. It is estimated that industrial by-products account for approximately 30% of total food production, leading to significant economic losses and environmental concerns [1]. In this context, the valorization of agro-industrial residues has gained increasing attention as a sustainable strategy to recover high-value compounds. Among these residues, fruit kernels represent a promising yet underexploited resource for the extraction of seed oils with potential applications in the food, pharmaceutical, and cosmetic sectors [2].

Vegetable kernel oils are recognized for their rich content of bioactive constituents, including unsaturated fatty acids (omega-3 and omega-6), phenolic compounds, tocopherols, and phytosterols, which collectively contribute to their nutritional, antioxidant, and functional properties [3]. The chemical composition and biological potential of these oils are strongly influenced by extraction methods [4]. Seeds with high oil yield, such as apricot kernels, are often subjected to cold pressing followed by solvent extraction, whereas low-oil-content seeds, including grape seeds, are typically processed directly with organic solvents. Although solvent extraction can achieve residual oil contents below 2%, this approach raises concerns related to solvent toxicity, high operational costs, and the degradation of thermolabile bioactive compounds during refining steps [5]. Consequently, cold extraction techniques are increasingly preferred for kernel oils, as they offer a more environmentally sustainable and economically viable process while preserving the native bioactive profile of the oil [6].

**Figure 1:**
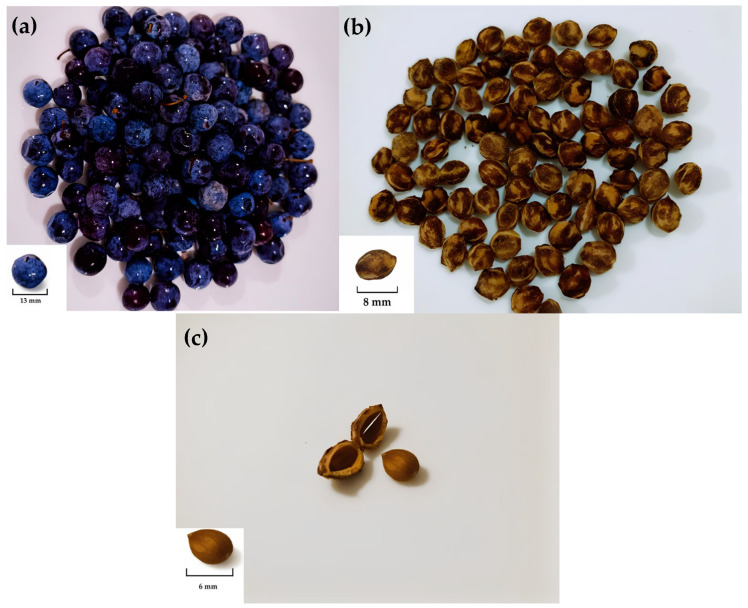
*Prunus spinosa*: fruit (**a**), kernel (**b**), and seed kernel (**c**).

Within this framework, growing interest has been directed toward oils derived from stone fruit kernels such as cherry, apricot, peach, and nectarine. However, sloe kernel oil remains largely unexplored, despite its potential value. *P. spinosa* is a wild Rosaceae species, commonly known as blackthorn or sloe, referred to as “*Barkouk lemaiz*” in Algeria and “*Lvarquq tɣeten*” in the Kabyle Berber language. The species originates from Western Asia and Northern Europe and is widely distributed across Mediterranean and northwestern African regions [7]. The plant produces small, spherical blue–black drupes with yellowish-green pulp characterized by pronounced astringent and acidic sensory attributes [8] (Figure 1).

Sloe fruits are well documented as a rich source of polyphenols, anthocyanins, flavonoids, vitamin C, and essential minerals such as calcium, magnesium, and potassium, conferring antioxidant, anti-inflammatory, antimicrobial, antidiabetic, and anticancer activities [9]. Traditionally, these fruits have been used to treat diabetes, oral inflammation, and gastrointestinal disorders. More recent studies have highlighted additional functional properties, including prebiotic effects, UV-protective capacity, and preservative applications in food systems [10]. By contrast, the kernel fraction and its oil have received comparatively limited attention, despite evidence indicating a favorable lipid profile. Previous analyses have reported high levels of oleic acid as the predominant fatty acid, followed by linoleic acid, along with appreciable amounts of γ-tocopherol, β-sitosterol, and aromatic compounds such as vanillin [11]. The remarkably high proportion of mono- and polyunsaturated fatty acids positions sloe kernel oil as a potentially valuable ingredient for functional and cosmetic formulations.

In the context of skin aging, increasing attention has been devoted to natural products capable of modulating enzymes involved in extracellular matrix degradation, such as matrix metalloproteinases, collagenase, and elastase, which play a central role in collagen and elastin breakdown [12,13]. In parallel, the incorporation of plant-derived oils into dermocosmetic formulations has been investigated not only for their emollient and occlusive properties but also for their ability to provide supportive photoprotective effects through UV absorption and antioxidant activity [14].

Against this background, the present study aims to provide a comprehensive characterization of *Prunus spinosa* kernel oil obtained by cold extraction, focusing on its physicochemical quality, chemical composition, and functional properties. Specifically, the study investigates the fatty acid profile, antioxidant capacity, and in vitro anti-aging enzyme activities of PSKO, and evaluates its incorporation into a moisturizing dermocosmetic formulation. In this context, the evaluation of natural oils for dermocosmetic applications should not be interpreted in terms of selective pharmacological inhibition, but rather as a functional modulation of multiple biological pathways involved in skin aging.

## 2. Results and Discussion

### 2.1. Oil Extraction and Yield

Cold-press extraction of 110 g of blackthorn kernel seeds obtained from 3 kg of fresh fruits yielded 39 g of oil, corresponding to an extraction yield of 35.45% ± 3.06. This value is comparable to those reported for other stone fruit kernels processed under similar conditions, such as sour cherry (30.0%), apricot (39.8%), and peach kernels (42.8) [1]. The relatively high oil yield highlights the potential of blackthorn kernels as an oleaginous raw material suitable for valorization. Despite being derived from a wild and underutilized species, *P. spinosa* kernels represent a promising source of oil that may be exploited for food, cosmetic, and pharmaceutical applications, particularly within sustainable and circular economy frameworks.

### 2.2. Levels of Polyphenols, Carotenoids, and Antioxidant Activity

Polyphenolic compounds contribute to oxidative stability by limiting lipid oxidation, while carotenoids provide both natural coloration and light-absorbing properties, which are of particular interest for dermocosmetic formulations. Given the limited literature available on blackthorn kernel oil, the characterization of these bioactive components is essential to define its functional profile. The analysis of *P. spinosa* kernel oil revealed a total phenolic content (TPC) of 70.37 mg GAE/kg oil (Table 1), which is markedly higher, approximately 21-fold, than the value reported by Vassilis et al. (3.33 mg GAE/kg). Such discrepancies may be attributed to differences in geographical origin, climatic conditions, and agronomic factors, which are known to influence the biosynthesis and accumulation of phenolic compounds in plant matrices. When compared with other fruit kernel oils, blackthorn kernel oil exhibited a higher phenolic content than apricot kernel oil (27.2 mg GAE/kg; [15], while remaining lower than peach kernel oil (170 mg GAE/kg; [16] and blueberry seed oil (89 mg GAE/kg; [17]. These comparisons position *P. spinosa* kernel oil within an intermediate-to-high range of phenolic content among fruit-derived oils. The total carotenoid content (TCC) reached 245.67 mg CtE/kg oil, closely matching previously reported values for blackthorn kernel oil (218.62 mg CtE/kg; [9]), thereby confirming good analytical reproducibility. Notably, this carotenoid level was substantially higher than those reported for sweet cherry (*Prunus avium*, 40.78 mg CtE/kg) and sour cherry (*Prunus cerasus* L., 84 mg CtE/kg) [18]), indicating that blackthorn kernel oil represents one of the richest carotenoid sources within the *Prunus* genus. The antioxidant activity, expressed as 89.35 mg AAE/kg oil, indicates a relevant free radical scavenging capacity. This activity is consistent with the elevated levels of phenolic compounds and carotenoids and likely reflects their synergistic contribution to antioxidant mechanisms in the oil matrix.

### 2.3. Quality Parameters

Lipid oxidation is one of the main deterioration pathways affecting vegetable oils, leading to the formation of primary and secondary oxidation products that negatively impact sensory quality, shelf life, and, in some cases, safety due to the generation of potentially harmful compounds such as aldehydes [19]. Therefore, the evaluation of oxidative stability parameters represents a critical step in defining oil quality and suitability for food and cosmetic applications. To assess the oxidative status of *Prunus spinosa* kernel oil, key physicochemical parameters were determined, including acidity value, peroxide value, specific extinction coefficients (K_232_ and K_270_), and refractive index (Table 2). The measured acidity value of 1.05 mg KOH/g oil indicates a low level of free fatty acids, reflecting good raw material quality and appropriate processing conditions. This value remains well below the maximum limit of 4 mg KOH/g established by the Codex Alimentarius for virgin vegetable oils (CODEX STAN 210-1999), confirming acceptable freshness and minimal hydrolytic degradation. The oil exhibited a very low peroxide value (0.19 meq O_2_/kg oil), indicative of minimal primary oxidation and excellent oxidative stability. This value is far below the maximum limit of 15 meq O_2_/kg specified for virgin oils (CODEX STAN 210-1999) and is notably lower than previously reported values for blackthorn kernel oil (1.48 meq O_2_/kg; [9]), suggesting improved preservation of the lipid fraction. Spectrophotometric analysis revealed a K_232_ value of 1.12, indicating a limited presence of conjugated dienes, which are typical markers of early-stage lipid oxidation. This result is consistent with values reported for other fresh kernel oils, such as plum and apricot oils [20]. The K_270_ value of 0.09 further confirms the negligible formation of secondary oxidation products, such as conjugated trienes and aldehydic compounds, and is considerably lower than values reported for other fruit seed oils, including pomegranate and grape seed oils [21]. The calculated K_232_/K_270_ ratio (12.4) indicates a clear predominance of primary oxidation products over secondary ones, supporting the overall assessment of high oxidative quality and minimal degradation. Finally, the refractive index (1.47) falls within the typical range reported for common vegetable oils such as corn, sesame, and sunflower oils [22], confirming the homogeneity of the lipid fraction and excluding potential adulteration. Overall, these parameters collectively demonstrate the high physicochemical quality and oxidative stability of *P. spinosa* kernel oil, supporting its suitability for dermocosmetic and functional applications.

### 2.4. GC-MS Profiling

GC–MS analysis of PSKO revealed a lipid profile largely dominated by unsaturated fatty acids (84.70%), with oleic acid (C18:1, ω-9) identified as the major component (65.89%). This value is comparable to that reported by Ilker et al. [11] (72.72%) and slightly higher than those observed in other studies, including [23] (48.83%), [9] (29.35%), and [24] (2025) (59.5%). Such variability is likely attributable to differences in geographical origin, climatic conditions, and genetic variability, which are known to influence lipid biosynthesis in fruit kernels. It should be noted that our samples were collected from wild trees growing at 700 m above sea level under full sun exposure in a Mediterranean semi-arid climate without any agricultural management. Among the cited studies, only Vassiliki et al. [9] provided geographical coordinates, indicating a collection site in Thessaly, Greece, at 400 m altitude. The other studies reported only the country of origin without specifying environmental conditions, altitude, or cultivation status. This lack of comparable environmental data severely limits the possibility of attributing the observed compositional differences to specific factors. Linoleic acid (C18:2, ω-6) accounted for 18.81% of the total fatty acid composition, representing an intermediate value within the wide range reported in the literature (Table 3). From a dermocosmetic perspective, this fatty acid composition offers several functional advantages. The high oleic acid content enhances skin penetration, provides emollient properties, and exhibits anti-inflammatory effects on cutaneous tissue. Linoleic acid contributes to epidermal barrier integrity by promoting ceramide synthesis and reducing transepidermal water loss, properties that are essential for skin hydration and barrier repair formulations [25]. The high proportion of mono- and polyunsaturated fatty acids observed in PSKO is consistent with profiles reported for several kernel oils and supports its relevance for nutritional and dermocosmetic applications, particularly in relation to skin barrier function and emollient properties [26,27]. In addition to these expected components, the present analysis revealed the presence of lauric acid (C12:0, 13.66%), which has not been previously reported for *P. spinosa* kernel oil. Although lauric acid is a saturated fatty acid, its presence may contribute to the oxidative stability and structural properties of the oil. This saturated fatty acid exhibits notable antimicrobial activity against Cutibacterium acnes and *Staphylococcus aureus* [28], making PSKO particularly suitable for formulations targeting acne-prone skin. Lauric acid also contributes to the oxidative stability of cosmetic emulsions and enhances skin moisturization [29]. Moreover, minor volatile compounds such as benzaldehyde (1.55%) and 1-butene, 4-isothiocyanato- (1.09%) were detected. While present in lower concentrations, these compounds may contribute additional functional properties: benzaldehyde possesses antimicrobial activity [30], while isothiocyanates activate Nrf2-mediated antioxidant pathways in skin cells and offer potential photoprotective effects against UV-induced oxidative stress [31]. These compounds, known for their aromatic characteristics and reported bioactivities in other plant matrices [32], may influence the sensory profile and functional properties of the oil. Overall, the GC–MS profile highlights the chemical complexity and variability of PSKO and supports its potential as an underexplored kernel oil for functional and dermocosmetic applications, while warranting further investigation into the role of minor constituents. Taken together, the newly detected lauric acid and 1-butene, 4-isothiocyanato- highlight additional bioactive relevance of PSKO, supporting its functional and dermocosmetic applications.

### 2.5. In Vitro Inhibition of MMP-9 Activity by PSKO

Vegetable oils, as complex lipid matrices, may interact with in vitro enzymatic assays through multiple mechanisms beyond direct enzyme inhibition, including altered substrate accessibility, sequestration of metal cofactors, and contributions from minor bioactive constituents [33] such as phenolics (70.37 ± 0.73 mg GAE/kg) and carotenoids (245.67 ± 2.89 mg CtE/kg) present in PSKO. Therefore, the observed effects should be interpreted as indicative of dermocosmetic rather than pharmacological activity, with biological relevance requiring validation through ex vivo or in vivo studies.

The inhibitory activity of *Prunus spinosa* kernel oil against MMP-9 was evaluated using a fluorometric enzymatic assay. PSKO induced a concentration-dependent reduction in MMP-9 activity across the tested concentration range (0.78–100 µg/mL), whereas the vehicle control (1% DMSO) did not significantly affect enzyme activity. At the highest tested concentration (100 µg/mL), PSKO inhibited MMP-9 activity by approximately 70–80%, with progressively lower inhibition observed at decreasing concentrations. Nonlinear regression analysis of the dose–response curve yielded an IC_50_ value in the low-to-mid µg/mL range (approximately 15–25 µg/mL), indicating a relevant inhibitory effect for a complex natural oil matrix. The reference inhibitor GM6001 (10 µM) produced near-complete inhibition (>90%), confirming the validity of the assay. PSKO background controls (oil + substrate, no enzyme) showed negligible fluorescence changes, excluding interference from intrinsic oil fluorescence or substrate quenching and confirming that the observed inhibition was attributable to genuine enzymatic modulation. Comparable inhibitory profiles have been described for cold-pressed oils such as olive, argan, and pomegranate seed oils, where partial MMP inhibition has been attributed to the combined action of unsaturated fatty acids and minor lipophilic antioxidants rather than to single dominant inhibitors [34,35]. The relatively higher inhibitory potency of PSKO toward MMP-9, compared with the other tested enzymes, may reflect a greater accessibility of the MMP-9 catalytic domain to lipid-derived modulators and supports a functional role of PSKO in the modulation of extracellular matrix remodeling processes relevant to skin aging. The moderate and concentration-dependent MMP-9 modulation observed for PSKO is comparable to that reported for other cold-pressed oils such as olive, argan, and pomegranate seed oils, which have been proposed as supportive anti-aging ingredients rather than pharmacological inhibitors.

### 2.6. Inhibition of Collagenase Activity

PSKO also exhibited a dose-dependent inhibitory effect on collagenase activity. Unlike polyphenol-rich extracts, lipid-based systems such as PSKO are not expected to act as direct collagenase. At 100 µg/mL, collagenase inhibition reached approximately 60–70%, while lower concentrations resulted in progressively reduced inhibition. The calculated IC_50_ value was in the mid-µg/mL range (approximately 25–35 µg/mL), indicating moderate but biologically relevant inhibitory potency. The positive control epigallocatechin gallate (EGCG) produced >85% inhibition, confirming assay reliability. Background controls showed negligible fluorescence interference, validating the enzymatic origin of the observed inhibition. The moderate but consistent inhibition of collagenase activity by PSKO is in agreement with previous observations reported for plant-derived oils and lipid-rich cosmetic ingredients, which typically display partial modulation of collagen-degrading enzymes within comparable concentration ranges. In oil-enriched emulsions, collagenase modulation may be associated with improved barrier function and lipid organization rather than direct enzyme blockade [36]. In these systems, collagenase inhibition is generally associated with the presence of unsaturated fatty acids and minor phenolic constituents capable of interacting with enzyme surfaces without inducing complete catalytic blockade. Moderate collagenase inhibition similar to that observed for PSKO has been reported for lipid-based cosmetic formulations, where controlled enzyme modulation contributes to wrinkle-preventive effects without altering physiological collagen turnover [37].

### 2.7. Inhibition of Elastase Activity

In the elastase inhibition assay, PSKO demonstrated a concentration-dependent reduction in elastase activity, albeit with lower potency compared to MMP-9 and collagenase (Table 4). At the highest concentration tested (100 µg/mL), elastase inhibition reached approximately 45–55%, with an estimated IC_50_ value in the 40–60 µg/mL range.

The reference inhibitor elastatinal almost completely suppressed elastase activity (>90%), whereas vehicle-treated controls showed no significant inhibition. Elastase is generally less sensitive to inhibition by plant-derived oils compared with metalloproteinases, with reported effects being moderate and concentration-dependent [38]. In the context of cosmetic formulations, this limited yet measurable elastase modulation may still contribute to the preservation of elastic fiber integrity over prolonged use, supporting a mild and well-tolerated anti-aging strategy rather than an aggressive enzymatic blockade. Within this framework, the modest elastase inhibition observed for PSKO should be interpreted as part of a broader, balanced anti-aging profile.

### 2.8. Cream Quality Analysis

Statistical analysis performed using Student’s *t*-test (Table 5) revealed significant differences between the blackthorn kernel oil-enriched cream and the control formulation for most evaluated parameters, indicating that the incorporation of *Prunus spinosa* kernel oil influenced the physicochemical characteristics of the emulsion.

The pH of the oil-enriched cream (5.53 ± 0.02) falls within the slightly acidic range compatible with healthy human skin, which is commonly reported to be between 4.5 and 5.5 and is important for maintaining skin barrier integrity and microbial homeostasis [39]. In contrast, the control cream exhibited a significantly higher pH (6.63 ± 0.08), exceeding this optimal range and potentially affecting skin surface equilibrium. The pH decreased from 6.63 to 5.53, likely due to weak acids introduced with PSKO (acid value: 1.05 ± 0.05 mg KOH/g) and the dehydroacetic acid present in 0.6% Cosgard^®^. This shift brings the formulation closer to the physiological skin pH, suggesting improved skin compatibility. In unbuffered oil-in-water emulsions, the measured pH represents an apparent value reflecting the equilibrium between the aqueous phase and dispersed lipid droplets. As a consequence, such systems are particularly sensitive to the introduction of weak acidic components, even at moderate concentrations.

Peroxide index values differed significantly between formulations, with the PSKO-enriched cream showing a lower peroxide value (0.15 ± 0.01 meq O_2_/kg) compared with the control (0.48 ± 0.01 meq O_2_/kg). Although both values remain well below the threshold commonly considered acceptable for cosmetic products, this result suggests that the incorporation of blackthorn kernel oil may contribute to improved oxidative stability of the formulation [40]. Moisture content analysis revealed a slight but statistically significant decrease in the oil-enriched cream (59.73 ± 0.07%) compared with the control (62.6 ± 0.11%). This difference can be attributed to the increased lipid fraction in the formulation, which alters the water-to-oil ratio and may favor the formation of a more structured hydrolipidic film on the skin surface. The occlusivity test showed a marked increase in occlusive capacity for the PSKO-enriched cream (33.93 ± 0.18%) compared with the control (14.41 ± 0.10%). This enhanced occlusivity indicates a greater ability to form a semi-occlusive barrier on the skin, which is known to reduce transepidermal water loss and support skin hydration by limiting water evaporation. Physical stability tests demonstrated that both formulations remained stable, with no evidence of phase separation during the observation period, indicating that the incorporation of blackthorn kernel oil did not compromise the structural integrity of the emulsion system.

### 2.9. UV–Visible Absorption Properties

UV–visible spectrophotometric analysis showed that PSKO exhibits a broad absorption profile across both UVB and UVA regions. Higher absorbance values were observed in the UVB range (290–320 nm), followed by a gradual decrease toward the UVA region (320–400 nm). This absorption behavior is consistent with the presence of carotenoids and other minor chromophoric constituents identified in the oil, which are known to contribute to light absorption in the ultraviolet region. The PSKO-enriched cream displayed significantly higher UV absorbance compared with the control formulation, particularly in the UVB range. This finding indicates that the incorporation of PSKO effectively enhances the UV-absorbing capacity of the cosmetic matrix. In contrast, the control cream showed negligible absorbance throughout the analyzed UV range, confirming the contribution of PSKO to the observed photoprotective behavior. The UV–visible absorption profile of PSKO, characterized by higher absorbance in the UVB region with a gradual decrease toward UVA wavelengths, is consistent with the behavior reported for other carotenoid- and unsaturated lipid-rich vegetable oils [41]. Comparable UV attenuation and absorption behaviors have been described for raspberry seed and carrot seed oils when incorporated into cosmetic matrices [42]. In this context, the absorption profile observed for PSKO reflects an intrinsic photoprotective contribution typical of cold-pressed plant oils, supporting its role as a complementary UV-absorbing component in dermocosmetic formulations rather than as a standalone sunscreen agent.

### 2.10. In Vitro SPF Estimation

Based on the Mansur spectrophotometric method, PSKO demonstrated a measurable in vitro SPF value (approximately SPF 3–5 at 1% concentration), indicating a modest but relevant UVB absorption capacity. The PSKO-enriched cream exhibited a higher in vitro SPF value (approximately SPF 6–8) compared with the control formulation, reflecting the contribution of PSKO within the emulsion system. These SPF values should be interpreted as indicative of a supportive photoprotective role, rather than as a standalone sunscreen effect, and are appropriate for daily-care dermocosmetic formulations where UV-filtering activity complements other functional properties. The modest in vitro SPF values obtained for PSKO and the PSKO-enriched cream are in line with those reported for several vegetable oils widely used in cosmetic formulations, including olive, carrot seed, and raspberry seed oils, which typically exhibit SPF values ranging between 2 and 10 when assessed by spectrophotometric methods [42]. Such values are generally interpreted as indicative of a supportive photoprotective contribution rather than primary sunscreen efficacy. Accordingly, the SPF values observed for PSKO support its potential use as a complementary ingredient contributing to daily photoprotection within dermocosmetic products, without replacing approved UV filters.

### 2.11. Photostability

Following artificial UV irradiation (Table 6), PSKO retained more than 85–90% of its initial absorbance in both the UVB and UVA regions, indicating good photostability under the tested conditions. Similarly, the PSKO-enriched cream showed only minor absorbance reductions after irradiation (<15%), whereas the control cream exhibited no relevant spectral changes due to its low initial UV absorbance. These results suggest that PSKO maintains its UV-absorbing properties upon UV exposure and does not undergo rapid photodegradation, supporting its suitability as a stable functional ingredient in dermocosmetic formulations. In this context, PSKO may contribute to UV absorption and photostability within daily-care dermocosmetic formulations, complementing conventional UV filters and antioxidant systems rather than replacing them. The retention of UV-absorbing capacity following irradiation suggests that PSKO does not undergo rapid photodegradation under the tested conditions, supporting its suitability for incorporation into dermocosmetic formulations where stability under light exposure is essential. This behavior further supports the role of PSKO as a stable, supportive photoprotective ingredient in dermocosmetic formulations.

### 2.12. Sensory Evaluation

**Figure 2:**
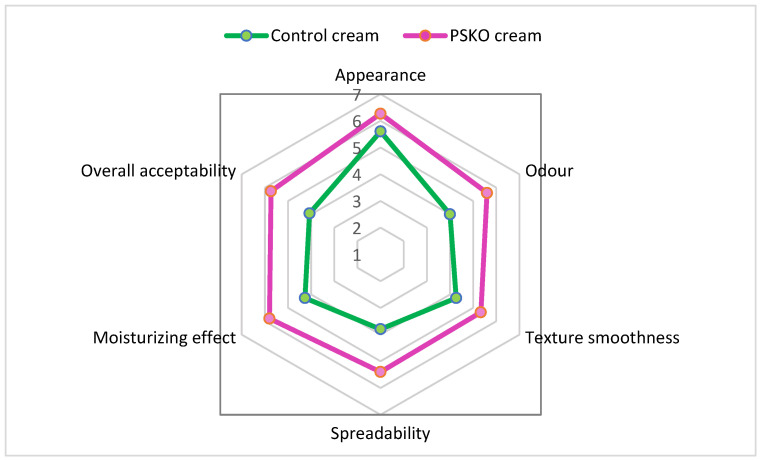
Radar chart of sensory descriptive analysis of control and PSKO-enriched cream formulations (scale 1–7).

The radar chart shown in Figure 2 summarizes the sensory descriptive analysis of the PSKO-enriched cream and the control formulation across six evaluated attributes. Overall, the PSKO-containing cream exhibited higher mean scores in most sensory parameters compared with the control, indicating a favorable impact of *Prunus spinosa* kernel oil incorporation on sensory perception. In particular, the PSKO-enriched formulation showed improved spreadability, texture smoothness, and immediate moisturizing effect, which are consistent with the higher lipid content and the physicochemical properties of the oil. Differences were also observed in the odor profile, with panelists reporting a more pleasant and characteristic aroma for the PSKO cream. This sensory feature may be partially associated with the presence of naturally occurring aromatic compounds, such as benzaldehyde, previously identified in the oil by GC–MS analysis, although the contribution of the overall formulation matrix cannot be excluded. The overall acceptability score was higher for the PSKO-enriched cream than for the control formulation, suggesting good consumer-oriented sensory performance. These findings indicate that the incorporation of blackthorn kernel oil can positively influence the sensory attributes of cosmetic emulsions without compromising formulation stability. Further formulation optimization, including the combination with additional functional ingredients, may allow fine-tuning of sensory properties and targeted performance depending on the intended dermocosmetic application. In the 24 h occlusive patch test, no adverse skin reactions were observed for either the PSKO-enriched cream or the control formulation. Visual assessment performed 30 min after patch removal showed no erythema or edema (score 0) at all application sites, and no participant reported itching, burning, or discomfort during the exposure period. These findings indicate good short-term skin tolerability. Overall, the integrated results support the valorization of *Prunus spinosa* kernel oil as a multifunctional dermocosmetic ingredient derived from an underutilized agri-food by-product, combining chemical stability, moderate biological activity, formulation compatibility, and supportive photoprotective properties within a sustainable circular economy framework.

Several limitations should be acknowledged. The sensory evaluation involved a limited panel size, and the 24 h patch test provides only preliminary tolerability data. Only a single oil concentration (10% *w*/*w*) was tested in the cream formulation; testing multiple concentrations would help optimize the balance between efficacy, sensory properties, and cost-effectiveness. Additionally, the in vitro enzyme inhibition and SPF results require further validation using ex vivo skin models and dermocosmetic application studies to confirm efficacy under real-use conditions and support photoprotection claims.

## 3. Materials and Methods

### 3.1. Reagents and Materials

All chemicals and reagents used in this study were of analytical or spectrophotometric grade. *n*-Hexane, methanol, ethanol, cyclohexane, ethyl acetate, diethyl ether, acetic acid, chloroform, potassium iodide, sodium thiosulfate, potassium hydroxide, phenolphthalein, DPPH, Tween-80, and dimethyl sulfoxide (DMSO, ≥99.5%) were purchased from standard commercial suppliers. Folin–Ciocalteu reagent, gallic acid, β-carotene, and ascorbic acid were used as reference standards for spectrophotometric assays.

Recombinant human matrix metalloproteinase-9, fluorogenic substrates, assay buffers, and reference inhibitors were obtained from commercially available fluorometric enzyme inhibition screening kits. Collagenase from *Clostridium histolyticum*, porcine pancreatic elastase, and their respective fluorogenic substrates and inhibitors were also sourced from validated commercial kits. All enzymatic assays were performed in black, flat-bottom 96-well microplates to minimize optical interference.

Commercial cosmetic base cream and Cosgard^®^ preservative were used for cream formulation. Ultrapure distilled water was used throughout all experiments. Analytical instruments included UV–Vis spectrophotometers, a GC–MS system, centrifuges, a pH meter, a refractometer, a moisture analyzer, and a microplate fluorescence reader, as specified in the corresponding methodological sections.

### 3.2. Cold Extraction of Blackthorn Kernel Oil

Fully ripe *Prunus spinosa* fruits were hand-harvested in September 2023 (early autumn) from wild plants in Minar Zareza, Mila region, Eastern Algeria (geographical coordinates: 36°31′33.7″ N, 5°52′38.4″ E). The plant material was botanically identified as *Prunus spinosa* L. (Rosaceae) by specialized agents from the Forest Conservation Department of Mila, who accompanied the authors during sampling, based on morphological characteristics and their expert knowledge of the species’ natural distribution. The sampling site is characterized by a Mediterranean semi-arid climate. The trees were growing naturally without any agricultural management (irrigation, fertilization, or chemical treatments). After collection, the fruits were thoroughly washed with distilled water and air-dried at ambient temperature (30 ± 5 °C, relative humidity 45 ± 5%) on perforated trays to reduce surface moisture. Once adequately dried, the fruits were manually pitted, and the kernels were carefully separated, sorted, and cleaned to remove residual pulp and foreign material. The kernels were then cracked to obtain the seeds, which were oven-dried at 40 °C for 3 h in a ventilated drying system (Memmert UF55, Schwabach, Germany). Oil extraction was performed by cold pressing using a continuous screw press (ZY-22A-01 Oil Press Machine, Ruian, China), operating below 40 °C to preserve thermolabile compounds. The crude oil was subsequently centrifuged to remove suspended particles, and the clear supernatant was collected and stored in amber glass bottles at 4 °C until further physicochemical and bioactive analyses.

### 3.3. Determination of Total Phenolic Content (TPC)

The extraction of total polyphenols from *Prunus spinosa* kernel oil was carried out following the protocol described by [43], with minor modifications. Briefly, 2.5 g of oil was mixed with 5 mL of *n*-hexane and 5 mL of a methanol/water solution (60:40, *v*/*v*). The mixture was vigorously vortexed for approximately 2 min to promote phase interaction and subsequently centrifuged at 3000 rpm for 10 min.

After centrifugation, the polar methanolic phase was carefully collected and immediately subjected to total phenolic content determination using the Folin–Ciocalteu colorimetric method, according to the procedure adapted by [44]. Absorbance was measured at 765 nm, and gallic acid was used as the reference standard. Results were expressed as milligrams of gallic acid equivalents per kilogram of oil (mg GAE/kg).

### 3.4. Determination of Total Carotenoid Content (TCC)

Total carotenoid content was determined according to the method described by [9]. Approximately 0.01 g of oil was dissolved in 5 mL of cyclohexane, and the absorbance of the resulting solution was measured at 450 nm using a 1 cm quartz cuvette.

Carotenoid concentration and extraction yield were expressed as β-carotene equivalents (CtE) and calculated using the following Equations (1) and (2):

(1) $${CC}_{n}=\frac{A_{450}\times DF}{A_{1\%}}\times C_{1\%}$$

(2) $${YC}_{n}=\frac{{CC}_{n}\times C_{1\%}}{M}$$

where CC_n_ represents the carotenoid concentration (µg/mL), A_450_ is the absorbance measured at 450 nm, DF is the dilution factor, A_1%_ corresponds to the specific absorbance of β-carotene in cyclohexane (2500), C_1%_ is equal to 10,000 µg/mL, V denotes the volume of cyclohexane (mL), YC_n_ is the extraction yield expressed as mg β-carotene equivalents per kg of oil (mg CtE/kg), and M represents the oil mass (kg).

### 3.5. DPPH Free Radical Scavenging Activity

Antioxidant activity of *Prunus spinosa* kernel oil was evaluated using the DPPH free radical scavenging assay, following the method described by [45] with minor adaptations. Briefly, 0.5 g of oil was homogenized with 5 mL of ethyl acetate to obtain a uniform solution. An aliquot of 50 µL of this extract was mixed with 950 µL of a freshly prepared DPPH solution.

After vigorous mixing, absorbance was measured at 515 nm immediately after mixing (A_0_) and after incubation for 30 min in the dark at room temperature (A_30_). The decrease in absorbance reflected the scavenging activity of the oil against the DPPH radical.

Antioxidant activity was calculated according to the following equation (Equation (3)) and expressed as ascorbic acid equivalents using a calibration curve (50–250 µg/mL):

(3) $$Antioxidant\;activity\%=\frac{\left(A_{0}-A_{30}\right)}{A_{30}}\;\times 100$$

where A_0_ represents the initial absorbance of the reaction mixture and A_30_ represents the absorbance after 30 min of incubation.

Antioxidant activity was expressed as ascorbic acid equivalents (AAE) using an ascorbic acid calibration curve in the concentration range of 50–250 µg/mL. Results were reported as mg AAE per kg of oil.

### 3.6. Determination of Oil Acidity

The acid value of *Prunus spinosa* kernel oil was determined according to the AOAC official method [46]. Briefly, 2.5 g of oil was dissolved in 50 mL of a diethyl ether/ethanol mixture (95:5, *v*/*v*) and titrated with 0.25 N potassium hydroxide using phenolphthalein as an indicator. The acid value was expressed as milligrams of potassium hydroxide required to neutralize the free fatty acids present in one gram of oil (mg KOH/g oil). All determinations were performed in triplicate.

### 3.7. Peroxide Value

Peroxide value was measured following the AOAC method [46] to assess the primary oxidation status of the oil. An oil sample (2.5 g) was mixed with chloroform and acetic acid, followed by the addition of potassium iodide solution. After reaction, distilled water was added, and the liberated iodine was titrated with 0.002 N sodium thiosulfate using starch as an indicator. Results were expressed as milliequivalents of active oxygen per kilogram of oil (meq O_2_/kg). Analyses were carried out in triplicate.

### 3.8. Refractive Index

The refractive index of the oil was measured at 25 °C using an Abbe refractometer (Model AR12, KRÜSS Optronic, Hamburg, Germany), in accordance with the AOAC method [46]. Measurements were performed in triplicate and used as an indicator of oil purity and homogeneity.

### 3.9. Determination of Specific Extinction Coefficients (K_232_ and K_270_)

Specific extinction coefficients at 232 nm (K_232_) and 270 nm (K_270_), corresponding to conjugated dienes and trienes, respectively, were determined following the method described by [47]. Briefly, 10 mg of oil was dissolved in 5 mL of cyclohexane, and absorbance was measured immediately using a UV–Vis spectrophotometer at the specified wavelengths with a 1 cm path-length quartz cuvette. These parameters were used to evaluate primary and secondary oxidation products. All measurements were conducted in triplicate.

### 3.10. GC-MS for Oil Composition

Prior to gas chromatographic analysis, fatty acid methyl esters (FAMEs) were prepared by transesterification of a 100 mg oil aliquot according to the method described by [48]. The resulting FAMEs were analyzed using a Shimadzu GCMS-QP2020 NX gas chromatograph coupled with a mass spectrometer (Shimadzu, Japan), equipped with an AOC-20s autosampler. Compound separation was achieved on an SH-I-5MS capillary column (30 m × 0.25 mm i.d., 0.25 µm film thickness). Helium was used as the carrier gas at a constant flow rate of 1.0 mL/min. Samples were injected in split mode (1:20) with an injection volume of 1 µL. The injector and interface temperatures were set at 200 °C and 300 °C, respectively. Fatty acid methyl esters were identified by comparing their retention times with those of reference standards and by matching mass spectra with data from the NIST mass spectral library.

### 3.11. In Vitro Enzyme Inhibition Assays

The experimental approach was adapted from previously validated fluorometric screening protocols for cosmetic enzyme modulation [49]. The MMP-9 inhibitory activity was evaluated using a fluorometric MMP-9 activity/inhibitor screening kit (Abcam, Cambridge, UK), according to the manufacturer’s instructions with minor adaptations. Collagenase inhibition was assessed using a fluorometric collagenase inhibitor screening kit (Sigma-Aldrich, St. Louis, MO, USA), while elastase inhibitory activity was determined using a fluorometric elastase inhibitor screening kit (Cayman Chemical, Ann Arbor, MI, USA).

#### 3.11.1. Preparation of PSKO Test Solutions

Due to the lipophilic nature of *Prunus spinosa* kernel oil, test solutions were prepared using a controlled dispersion approach to ensure reproducibility and minimize solvent-related effects. A primary stock solution of PSKO (200 mg/mL) was prepared in dimethyl sulfoxide (DMSO) by vigorous vortexing for 2 min followed by brief sonication (5 min, room temperature). This stock was subsequently diluted 1:100 in assay buffer containing 0.05% (*v*/*v*) Tween-80 to obtain an intermediate working solution (2 mg/mL). All final dilutions were prepared from this working solution using the corresponding assay buffer. The final concentration of DMSO was kept constant at 1.0% (*v*/*v*) in all wells, including samples and controls.

PSKO was tested over a concentration range of 0.78–100 µg/mL (eight-point, two-fold serial dilution), selected to provide reliable dose–response curves while limiting optical or matrix interference typical of lipid samples.

#### 3.11.2. MMP-9 Fluorometric Inhibition Assay

The inhibitory activity of PSKO against MMP-9 was evaluated using a commercial fluorometric MMP-9 activity/inhibitor screening kit, following the manufacturer’s instructions with minor adaptations. Assays were conducted in black, flat-bottom 96-well microplates at 37 °C, with a final reaction volume of 100 µL per well.

Each well contained PSKO dilution or vehicle control, recombinant human MMP-9 enzyme solution prepared at the recommended working concentration, and assay buffer. After a pre-incubation period of 15 min at 37 °C to allow interaction between PSKO and the enzyme, the reaction was initiated by adding the fluorogenic substrate. Fluorescence was monitored kinetically every 60 s for 30 min at excitation/emission wavelengths of 328/393 nm. Enzymatic activity was calculated from the linear portion of the fluorescence increase (ΔRFU/min).

A broad-spectrum metalloproteinase inhibitor (GM6001, 10 µM) was used as a positive control. Vehicle controls, blank wells (no enzyme), and PSKO background controls (oil + substrate, no enzyme) were included on each plate to account for non-enzymatic signal contribution and potential optical interference.

#### 3.11.3. Collagenase Inhibition Assay

Collagenase inhibitory activity was evaluated using a fluorometric assay based on *Clostridium histolyticum* collagenase and a specific fluorogenic peptide substrate. The assay was performed in black 96-well plates at a final volume of 100 µL per well.

PSKO test solutions were prepared as described above and evaluated at concentrations ranging from 0.78 to 100 µg/mL. After pre-incubation of PSKO or vehicle with the collagenase enzyme solution for 15 min at 37 °C, the reaction was initiated by adding the fluorogenic substrate. Fluorescence was recorded kinetically at excitation/emission wavelengths of 490/520 nm for 30 min, and enzyme activity was calculated from the linear reaction phase.

Epigallocatechin gallate (EGCG, 50 µM) was used as a reference inhibitor. Blank and PSKO background controls were included to verify the enzymatic origin of the observed inhibition.

#### 3.11.4. Elastase Inhibition Assay

Elastase inhibitory activity was determined using a fluorometric assay based on porcine pancreatic elastase and a specific elastase-sensitive fluorogenic substrate. The assay was conducted in black 96-well microplates at 37 °C with a final volume of 100 µL per well.

PSKO was tested over the same concentration range used for MMP-9 and collagenase assays (0.78–100 µg/mL). After pre-incubation of PSKO or vehicle with the elastase enzyme solution for 10 min at 37 °C, the reaction was initiated by addition of the fluorogenic substrate. Fluorescence was monitored at excitation/emission wavelengths of 400/505 nm for 30 min.

Elastatinal (10 µM) served as the positive control inhibitor, while vehicle and background controls were included in each experiment.

#### 3.11.5. Calculation of Enzyme Inhibition and IC_50_ Values

For all enzyme assays, fluorescence data were blank-corrected, and enzymatic activity was expressed as the slope of fluorescence increase (ΔRFU/min). Percentage inhibition was calculated relative to the vehicle-treated enzyme control. When necessary, background signals from PSKO optical controls were subtracted prior to inhibition calculation.

Dose–response curves were generated by plotting percentage inhibition against the logarithm of PSKO concentration, and IC_50_ values were estimated by nonlinear regression using a four-parameter logistic model. All measurements were performed in triplicate, and experiments were repeated in at least two independent runs. Results are reported as mean ± standard deviation. This experimental design, including the use of vehicle controls, background optical controls, and reference inhibitors, ensured the reliability and interpretability of enzyme inhibition data obtained from a complex lipid matrix such as PSKO.

### 3.12. Cream Preparation

The moisturizing cream was prepared using a commercially available semi-solid O/W (oil-in-water) base cream supplied as a concentrated emulsion. According to the supplier’s technical specifications, the base cream is primarily composed of purified water as the continuous aqueous phase, mineral oil (Paraffinum Liquidum) serving as the lipophilic component to prevent transepidermal water loss, ozokerite acting as a stabilizing agent in the emulsion system, and non-ionic vegetable-derived emulsifiers, including glyceryl stearate and polyglyceryl-3-oleate esters, which provide emulsion stability and impart a non-greasy, silky texture to the formulation. The hydrogenated castor oil present in the formulation functions as a co-emulsifier and surfactant, facilitating the incorporation of poorly water-soluble ingredients. The base cream exhibited a paste-like yet slightly fluid consistency, reflecting its high apparent viscosity and pseudoplastic rheological behavior typical of O/W emulsion systems. The composition is reported according to INCI nomenclature to the extent permitted by supplier disclosure.

Briefly, 200 mL of distilled water, preheated to 80 °C, was gradually added to 100 g of base cream under continuous mechanical stirring, following the supplier’s instructions, to achieve controlled dilution of the aqueous phase until a smooth, homogeneous emulsion free of visible granules was obtained. This dilution affected the viscosity of the system but did not modify the emulsion type or the interfacial organization of the O/W system. A preservative (Cosgard^®^, a synthetic blend of benzyl alcohol and dehydroacetic acid) was subsequently incorporated at a concentration of 0.6% (*w*/*w*). No buffering system was used.

The experimental formulation was enriched with *Prunus spinosa* kernel oil at 10% (*w*/*w*), while the control cream was prepared following the same procedure without oil addition. Both formulations were allowed to cool to room temperature under gentle stirring and were stored in airtight containers until further analyses. The prepared creams were subjected to physicochemical, functional, and sensory evaluations to assess the effect of PSKO incorporation.

### 3.13. Anti-Aging Moisturizing Cream Quality Assessment

Quality parameters were evaluated for both the PSKO-enriched cream and the control formulation. The pH was measured at room temperature using a calibrated digital pH meter (Edge^®^, HI-2002, Hanna Instruments, Woonsocket, RI, USA). Oxidative stability was assessed by determining the peroxide value according to the AOAC standard method.

Physical stability of the formulations was evaluated by centrifugation at 4000 rpm for 20 min using a Sigma 3-30 KS centrifuge (Sigma, Germany), followed by visual inspection for any signs of phase separation [50]. Moisture content was determined gravimetrically using an electronic moisture analyzer (Radwag MA 110.R, Radwag, Radom, Poland) at 120 °C.

The in vitro occlusivity test was performed according to the method described by Maru et al. [51]. Briefly, 200 mg of each cream sample were uniformly spread on filter paper (0.45 µm pore size) placed over glass beakers containing 10 g of distilled water. The beakers were sealed and maintained at 37 ± 2 °C and 60 ± 5% relative humidity for 48 h. Water loss was determined gravimetrically, and the occlusion factor was calculated by comparing water evaporation from uncovered beakers with that from sample-covered beakers.

### 3.14. In Vitro UV-Protective Properties

UV–Visible Absorption Spectra of PSKO and PSKO-Enriched Cream.

The UV-protective potential of PSKO and the PSKO-enriched cream was preliminarily evaluated by UV–visible spectrophotometry, following a previously described comparative approach [52].

For PSKO analysis, the oil was dispersed in ethanol (spectrophotometric grade) to obtain a 1% (*w*/*v*) dispersion. Although vegetable oils form colloidal dispersions rather than true solutions in ethanol, this standardized approach enables comparative UV absorption assessment. The UV–Vis absorption spectrum was recorded in the range 200–400 nm using a quartz cuvette (path length 1 cm), with ethanol as blank. Samples were homogenized by vortexing immediately before each measurement.

For the cream formulations, samples (1.0 g) were dispersed in 10 mL of ethanol under magnetic stirring for 30 min, followed by centrifugation (4000 rpm, 10 min). The clear supernatant was collected and analyzed under the same spectral conditions. The control cream (without PSKO) was analyzed in parallel.

Absorption profiles were evaluated separately for: UVB region: 290–320 nm; UVA region: 320–400 nm.

### 3.15. In Vitro SPF Determination

The in vitro sun protection factor (SPF) was estimated using the Mansur spectrophotometric method widely adopted for preliminary cosmetic screening [53]. Absorbance values were recorded at 5 nm intervals between 290 and 320 nm using a UV–visible spectrophotometer. SPF values were calculated by weighting the measured absorbance at each wavelength with the corresponding erythemal effect spectrum and solar intensity factors, applying a correction factor of 10. This approach provides a relative and comparative estimate of UVB protection and is widely used for preliminary evaluation of cosmetic formulations; however, it does not represent a clinical SPF determination

### 3.16. Photostability Assessment

Photostability of PSKO and PSKO-enriched cream was evaluated by comparing UV–Vis spectra before and after UV irradiation.

Samples were exposed to artificial UV light (UVA/UVB lamp, 365 nm dominant wavelength) for 2 h at a fixed distance (20 cm) under controlled temperature conditions (25 ± 2 °C). After irradiation, samples were reanalyzed spectrophotometrically under identical conditions.

Photostability was assessed by calculating the percentage variation of absorbance at selected wavelengths representative of UVB (300 nm) and UVA (340 nm) regions.

### 3.17. Sensory Analysis

Sensory evaluation of the cream formulations was conducted according to a modified protocol described by Bîrsan et al. [50], using a trained panel of 15 female assessors aged between 22 and 40 years. Panelists were selected based on prior experience with cosmetic sensory analysis and were familiarized with the evaluation protocol before testing. All evaluations were performed under controlled conditions in a dedicated sensory analysis room at ambient temperature.

The panel assessed six sensory attributes: appearance, odor, texture smoothness, spreadability, immediate moisturizing effect, and overall acceptability. Samples were coded with random three-digit numbers and presented in a randomized order to minimize bias. Approximately equal quantities of each formulation were applied to the volar forearm, and panelists were allowed sufficient time for evaluation and perception stabilization before scoring.

A seven-point hedonic scale was used for each attribute, where 1 corresponded to “extremely unsatisfactory” and 7 to “extremely satisfactory”. Individual scores were collected and used for statistical analysis. Results were expressed as mean values and graphically represented using radar charts to allow direct comparison between formulations. All panelists provided informed consent prior to participation (Document S1, Supplementary Material). The sensory evaluation was conducted for cosmetic research purposes only and did not involve any therapeutic or clinical claims.

### 3.18. Skin Tolerability—24 h Patch Test

A preliminary skin tolerability assessment of the cream formulations was performed using a 24 h occlusive patch test on healthy volunteers, according to a standardized cosmetic safety screening protocol [54]. A fixed amount of each formulation (approximately 0.20 g) was applied to separate sites on the volar forearm using occlusive patches (standard hypoallergenic adhesive patches). The PSKO-enriched cream and the control cream were applied in a randomized left/right allocation to minimize site-related bias. Patches were maintained in place for 24 h under normal daily activity conditions, avoiding washing of the application area. After patch removal, the test sites were gently inspected after a 30 min recovery period to reduce transient pressure-related erythema. Skin reactions were evaluated by visual scoring for erythema and edema using a semi-quantitative scale (0 = none, 1 = slight, 2 = moderate, 3 = marked) (Table S1, Supplementary Material). Participants were instructed to report any subjective symptoms (itching, burning, discomfort) during the exposure period.

### 3.19. Statistical Analysis

All experiments were performed in triplicate unless otherwise stated, and results are expressed as mean ± standard deviation (SD). Enzymatic inhibition assays were conducted using technical triplicates for each concentration, and experiments were repeated in at least two independent runs to ensure reproducibility. Dose–response curves and half-maximal inhibitory concentration (IC_50_) values were obtained by nonlinear regression using a four-parameter logistic model.

Statistical comparisons between formulations or treatments were performed using one-way analysis of variance (ANOVA), followed by an appropriate post hoc test when applicable. For sensory evaluation and cream quality parameters, Student’s *t*-test was used to compare PSKO-enriched and control formulations. Differences were considered statistically significant at *p* < 0.05. All statistical analyses were performed using GraphPad Prism software (version 10.0 or later; GraphPad Software, San Diego, CA, USA). Overall, these findings support the valorization of *Prunus spinosa* kernel oil as a sustainable, bioactive ingredient derived from an underutilized agro-food by-product for innovative dermocosmetic applications.

## 4. Conclusions

This study provides a comprehensive evaluation of *Prunus spinosa* kernel oil as an underexplored natural resource with potential relevance for dermocosmetic applications. Cold extraction of blackthorn kernels yielded an oil characterized by a favorable fatty acid profile dominated by unsaturated fatty acids, together with appreciable levels of phenolic compounds and carotenoids. The oil exhibited good physicochemical quality and oxidative stability, supporting its suitability for incorporation into cosmetic formulations.

Functional investigations demonstrated that PSKO is capable of modulating key extracellular matrix-degrading enzymes involved in skin aging, including matrix metalloproteinase-9, collagenase, and elastase, with a concentration-dependent inhibitory effect. Although moderate in magnitude, this multi-target enzymatic modulation is noteworthy for a complex natural oil matrix and supports its role as a functional cosmetic ingredient rather than as a single-compound active.

The incorporation of PSKO into a moisturizing cream resulted in improved physicochemical properties, including a skin-compatible pH, enhanced occlusive capacity, and maintained formulation stability. Sensory evaluation further indicated favorable consumer-oriented attributes, with higher overall acceptability compared with the control formulation. In addition, UV–visible spectrophotometric analyses revealed that both PSKO and the PSKO-enriched cream exhibit supportive UV-absorbing properties and good photostability, contributing to a complementary photoprotective effect suitable for daily-care dermocosmetic products.

Overall, these findings highlight *Prunus spinosa* kernel oil as a promising natural ingredient that combines chemical stability, functional bioactivity, and formulation performance. Future studies should focus on long-term stability, skin penetration behavior, and in vivo evaluation to further define its role within advanced dermocosmetic formulations.

## Figures and Tables

**Table 1:** Total phenolic content, carotenoid content, and antioxidant activity of *Prunus spinosa* kernel oil.

Parameter	Value
TPC (mg GAE/kg oil)	70.37 ± 0.73
TCC (mg CtE/kg oil)	245.67 ± 2.89
Antioxidant activity (mg AAE/kg oil)	89.35 ± 0.65

Data are expressed as mean ± SD (n = 3). TPC: total phenolic content, expressed as gallic acid equivalents (GAE); TCC: total carotenoid content, expressed as β-carotene equivalents (CtE); AA: antioxidant activity, expressed as ascorbic acid equivalents (AAE).

**Table 2:** Oxidative stability and quality parameters of *Prunus spinosa* kernel oil.

Parameter	Value
Acidity (mg KOH/g oil)	1.05 ± 0.05
Peroxide value (meq O_2_/kg oil)	0.19 ± 0.02
Refractive index	1.47 ± 0.00
K_232_	1.12 ± 0.03
K_270_	0.09 ± 0.01

Data are expressed as mean ± SD (n = 3). K_232_ and K_270_ represent the specific extinction coefficients at 232 and 270 nm, respectively, commonly used to assess primary and secondary lipid oxidation products.

**Table 3:** GC–MS profile of fatty acids and volatile compounds identified in *Prunus spinosa* kernel oil, with comparison to literature data.

Compound	This Study (%)	[23]	[11]	[9]	[24]
Oleic acid (C18:1, ω-9)	65.89	48.83	72.72	29.35	59.5
Linoleic acid (C18:2, ω-6)	18.81	41.16	17.73	64.4	9.6
Lauric acid (C12:0)	13.66	–	–	–	–
Benzaldehyde	1.55	–	–	–	8.2
1-Butene, 4-isothiocyanato-	1.09	–	–	–	–
Total saturated fatty acids	13.66	8.34	8.40	6.04	9.2
Total unsaturated fatty acids	84.70	91.66	91.60	93.96	69.1
Saturated/unsaturated ratio	0.16	0.09	0.09	0.06	0.13
Geographical origin	Algeria	Turkey	Turkey	Greece	Serbia

Data are expressed as relative percentage composition (% of total identified compounds). “–” indicates not reported. Differences among studies may reflect variations in cultivar, geographical origin, climatic conditions, and analytical methodologies.

**Table 4:** In vitro inhibition of extracellular matrix-degrading enzymes by *Prunus spinosa* kernel oil.

Enzyme	Inhibition at 100 µg/mL (%)	IC_50_ (µg/mL)	Positive Control (Inhibition %)
MMP-9	70–80	15–25	GM6001 (>90%)
Collagenase	60–70	25–35	EGCG (>85%)
Elastase	45–55	40–60	Elastatinal (>90%)

Data are expressed as mean values from at least three independent measurements. IC_50_ values were estimated by nonlinear regression analysis. GM6001: Galardin; EGCG: epigallocatechin gallate.

**Table 5:** Statistical comparison of physicochemical quality parameters of control and PSKO-enriched cream formulations.

Quality Parameter	PSKO-Enriched Cream	Control Cream	*p*-Value
pH	5.53 ± 0.02	6.63 ± 0.08	0.0011 *
Peroxide value (meq O_2_/kg oil)	0.15 ± 0.01	0.48 ± 0.01	<0.0001 *
Moisture content (%)	59.73 ± 0.07	62.60 ± 0.11	<0.0001 *
Occlusivity (%)	33.93 ± 0.18	14.41 ± 0.10	<0.0001 *
Physical stability	Stable (no phase separation)	Stable (no phase separation)	–

Data are expressed as mean ± SD (n = 3). Statistical significance was determined using Student’s *t*-test. * *p* < 0.05 was considered statistically significant.

**Table 6:** In vitro UV-protective properties and photostability of PSKO and PSKO-enriched cream.

Sample	UVB Absorbance (290–320 nm)	UVA Absorbance (320–400 nm)	In Vitro SPF (Mansur Method)	Residual UV Absorbance After Irradiation (%)	Photostability
PSKO (1% *w*/*v* in ethanol)	High	Moderate	4.3 ± 0.4	88.6 ± 2.1	Good
PSKO-enriched cream (10% *w*/*w*)	Moderate–High	Moderate	7.1 ± 0.6	85.2 ± 2.8	Good
Control cream (without PSKO)	Low	Negligible	<1	n.a.	n.a.

UV absorbance values were obtained from UV–Vis spectra recorded between 200 and 400 nm. In vitro SPF values were calculated using the Mansur spectrophotometric method (CF = 10). Photostability was evaluated by comparing absorbance at representative UVB (300 nm) and UVA (340 nm) wavelengths before and after 2 h of artificial UV irradiation. Data are expressed as mean ± SD (n = 3). n.a.: not applicable due to negligible initial absorbance.

## Data Availability

The original contributions presented in the study are included in the article/Supplementary Materials. Further inquiries can be directed to the corresponding author.

## Supplementary Materials

The following supporting information can be downloaded at: https://www.mdpi.com/article/10.3390/molecules31040632/s1. Document S1: Informed Consent Form for Sensory Evaluation and Skin Tolerability Assessment; Table S1: Results of the 24 h occlusive patch test for skin tolerability of cream formulations.
