# Supplementary material for: Physicochemical Characterization, In Vitro Anti-Aging Enzyme Modulation, and Dermocosmetic Application of Prunus spinosa L. Kernel Oil
Source: Molecules. 2026 Feb 12;31(4):632. doi: 10.3390/molecules31040632 (PMC12943284; doi:10.3390/molecules31040632)
Supplement: Supplementary file 1 [file molecules-31-00632-s001.zip › molecules-4120745-supplementary.pdf]

Supplementary Document S1.
Informed Consent Form for Sensory Evaluation and Skin Tolerability Assessment

Study Title:
Sensory Evaluation and Skin Tolerability Assessment of a Cosmetic Cream Formulated with *Prunus spinosa* L. Kernel Oil

Purpose of the Study:
You are invited to participate in a cosmetic research study conducted for scientific purposes. The aim of this study is to evaluate the sensory characteristics (appearance, odour, texture, spreadability, moisturizing effect, and overall acceptability) and the short-term skin tolerability of cosmetic cream formulations intended for dermocosmetic applications.

Procedures
If you agree to participate, you will be asked to take part in two non-invasive procedures:
Sensory evaluation:

- A small amount of cosmetic cream will be applied to the volar forearm, and you will be asked to evaluate its sensory properties using a structured scoring scale. This evaluation will take place under controlled conditions and will require approximately 10–15 minutes.
- Skin tolerability assessment (24 h patch test): a fixed amount of the cosmetic cream will be applied to a small area of the volar forearm using an occlusive patch, which will remain in place for 24 hours. After patch removal, the application site will be visually inspected for any signs of skin reaction.

Risks and Discomforts:
The tested products are cosmetic formulations intended for topical use only. No significant risks are anticipated. In the unlikely event of skin irritation, redness, itching, burning sensation, or discomfort, you may remove the product immediately and discontinue participation without any consequences.

Benefits:
There are no direct benefits to you from participating in this study. However, your participation will contribute to scientific research on the development, evaluation, and safety screening of cosmetic formulations.

Voluntary Participation:
Your participation in this study is entirely voluntary. You may withdraw from the study at any time, without providing a reason and without any negative consequences.

Confidentiality:
All information collected during the study will be treated as confidential. Data will be analyzed and reported only in aggregated form, and no personally identifiable information will be disclosed.

Ethical Considerations:
This study was conducted exclusively for cosmetic research purposes and does not involve therapeutic, diagnostic, or clinical claims.

Consent Statement:
By signing below, you confirm that you have read and understood the information provided above and that you voluntarily agree to participate in this sensory evaluation and skin tolerability study.

Participant Name (printed): ____________________________

Signature: ____________________________ Date: ____________

Supplementary Table S2. Results of the 24 h occlusive patch test for skin tolerability of cream formulations.

**Parameter**	**PSKO-enriched cream**	**Control cream**
Number of participants (n)	15	15
Applied dose per site (g)	0.20	0.20
Exposure time (h)	24	24
Erythema score (0–3)*	0.00 ± 0.00	0.00 ± 0.00
Edema score (0–3)*	0.00 ± 0.00	0.00 ± 0.00
Participants reporting itching/burning (n)	0	0
Participants with any adverse reaction (n)	0	0

*Scoring scale: 0 = none; 1 = slight; 2 = moderate; 3 = marked. Data are expressed as mean ± SD.
